# A Dual-Branch Artifact-Resistant Network for Air-Coupled Ultrasonic Localization Imaging of Composite Damage

**DOI:** 10.3390/s26216745

**Published:** 2026-10-25

**Authors:** Da Kang, Xiaohan Li, Zhenggan Zhou, Jielin Wang, Yuan Liu

**Affiliations:** 1 School of Mechanical Engineering and Automation, Beihang University, Beijing 100083, China; kang_dandt@buaa.edu.cn (D.K.); zzhenggan@buaa.edu.cn (Z.Z.); 2 Beijing Power Machinery Institute, Beijing 100074, China; 3 School of Instrumentation Science and Opto-Electronic Engineering, Nanchang Hangkong University, Nanchang 330063, China; 13969136106@163.com (X.L.); 2408085407335@stu.nchu.edu.cn (J.W.); 4 Key Laboratory of Nondestructive Testing (Ministry of Education), School of Instrumentation Science and Opto-Electronic Engineering, Nanchang Hangkong University, Nanchang 330063, China

**Keywords:** composite materials, air-coupled ultrasound, lamb wave, dual-branch convolutional neural network, artifact-resistant imaging

## Abstract

Composite materials are prone to low-velocity impact during service. This impact causes invisible internal damage and threatens structural safety. Air-coupled ultrasonic Lamb wave testing offers non-contact advantages. However, line scanning sparsity and anisotropic noise challenge traditional imaging methods. These challenges affect reconstruction efficiency and anti-artifact capability. To address this, this paper proposes a Dual-branch Artifact-Resistant Fast Imaging Network (DARF-Net). The network achieves end-to-end localization and reconstruction of composite damage. The method performs line scans along 0° and 90° directions. It feeds Lamb wave signals from two orthogonal directions into independent encoding branches. This extracts dual-view complementary features. The bottleneck layer uses element-wise addition fusion. It integrates dual-view information, enhances true damage responses, and suppresses noise. Residual blocks are embedded in the decoder to improve gradient propagation and reconstruction details. The network output undergoes morphological post-processing to suppress residual artifacts. Experiments use composite plate samples. Results show a mean center positioning deviation of 0.133 mm. The mean absolute diameter deviation is 0.188 mm. The method maintains high localization accuracy for damage offset 10 mm from the training grid. Single-image inference takes 14.79 ms. Validation is performed on calibrated surrogate damage produced by coupled mass blocks. The proposed method balances imaging accuracy and inference efficiency. It provides a feasible solution for rapid field evaluation of composite structures.

## 1. Introduction

Composite materials are widely used in engineering applications. Carbon fiber reinforced polymer (CFRP) offers excellent specific strength, specific stiffness, and corrosion resistance. These properties enable widespread applications in aerospace, rail transit, and new energy fields [1,2,3,4,5]. These fields have an urgent demand for lightweight structures. However, CFRP laminates are susceptible to random loads during service. A typical example is low-velocity impact. This impact induces internal damage, such as delamination and matrix cracking. This damage is invisible on the surface. It significantly reduces the load-bearing capacity and shortens the service life of the structure [6]. Therefore, fast and accurate nondestructive testing (NDT) techniques are needed. They should identify and localize internal damage in CFRP. This is an urgent need for engineering safety assurance. It is also a continuous research focus in the NDT field. Structural health monitoring (SHM) of composite structures aims at a continuous in-service assessment of structural integrity, whereas nondestructive testing (NDT) provides periodic off-line inspection with a higher spatial resolution. The statistical variability of guided-wave features in CFRP components has been quantified in recent SHM studies [7], which is one of the reasons why the present method learns the mapping from the measured response to the damage position directly instead of relying on a fixed threshold on a hand-crafted damage index.

Air-coupled ultrasonic guided wave technology uses air as the coupling medium. When an ultrasonic transducer emits a sound beam into a plate-like structure at a specific inclination angle, it excites specific Lamb wave modes in the plate. This technology combines the advantages of non-contact detection [6,8] and the high sensitivity of Lamb waves to deep damage [9]. In terms of detection capability, Felix et al. [10] introduced the MUSIC algorithm to eliminate blind zones in non-contact detection. Experimental results demonstrated the excellent sensing capability of Lamb waves for defects. Liu et al. [11] combined air-coupled Lamb wave scanning with a virtual time reversal algorithm. This achieved baseline-free detection of delamination defects in composite plates without relying on healthy baseline signals. In terms of quantitative accuracy, Bustamante et al. [12] compared laser-air-coupled and pure air-coupled methods. They found that the measured defect sizes agreed well with the designed values. This confirmed the accuracy and reliability of the detection. Yu et al. [9] used a cross-correlation damage detection technique. It can not only detect delamination defects but also accurately quantify the location and size of the damage. The above works validated the effectiveness and reliability of Lamb waves in composite damage assessment from two dimensions: detection capability and quantitative accuracy. However, relying solely on the observation of Lamb wave signal changes makes it difficult to directly determine the location and geometric size of damage. Imaging methods are needed to visualize damage features. This supports rapid and accurate detection decisions.

Visualization of Lamb wave detection results is usually achieved by C-scan or line scan modes. Traditional C-scan acquires data point by point. Its detection efficiency is low [13]. Complete scanning and processing of large components can take up to 6 h [14]. Line scan greatly reduces sampling points. It improves detection throughput. It is more suitable for rapid inspection of large-area composite materials. Toyama et al. [15] first applied line scan technology to CFRP delamination detection. They measured the arrival time and amplitude variation in the S_0_ mode. This achieved detection and localization of impact-induced delamination. Lee et al. [16] extended this method to online inspection scenarios. They used laser excitation and air-coupled reception. They achieved non-contact online detection of CFRP delamination defects through line scan frequency features. Tian et al. [17] addressed the insufficient detection accuracy for small defects. They proposed a nonlinear defect index and an adaptive weighted imaging algorithm. The area detection error for a 10 mm defect was only 7.8%. Xiao et al. [18] further extended air-coupled Lamb wave tomography from flat plates to curved CFRP laminates. This expanded the application of line scan modes in complex geometric structures. The above methods [17,18] still rely on manually designed damage indices or fixed wave velocity models. Under sparse data conditions, prior parameters are difficult to estimate accurately. This leads to a significant decrease in imaging resolution and localization accuracy. A new method is urgently needed. It should automatically learn the damage feature mapping relationship from sparse data. It should not rely on precise physical models.

Deep learning methods offer automatic feature extraction and end-to-end mapping. These advantages provide a new solution path for rapid imaging of line scan data. In early studies, Duan et al. [19] used a one-dimensional CNN to detect impact damage from air-coupled ultrasonic A-scan signals. This validated the feasibility of deep learning for extracting damage features from raw signals. Schmid et al. [20] systematically investigated neural network architectures and training strategies for both A-scan and C-scan data types. They revealed the potential of spatiotemporal information fusion in air-coupled ultrasonic testing. On this basis, researchers further applied deep learning to end-to-end imaging tasks. Luo et al. [21] constructed a Bayesian neural network to achieve damage detection and localization in CFRP plates. Probabilistic modeling effectively enhanced robustness to noise. Liu et al. [22] designed a multi-scale interactive Siamese network. It improved the line scan imaging efficiency by about 168.9% compared to traditional C-scan. Zhang et al. [23] proposed a UNet based on convolutional sparse coding (CSCUNet). It reconstructed low-resolution delay-and-sum images into high-resolution damage images. This achieved joint evaluation of the location, size, and contour of delamination damage. However, the above end-to-end methods only input line scan data from a single direction. They do not incorporate cross-localization information from orthogonal directions into the imaging decision. Furthermore, some studies attempt to suppress noise interference at the data and feature levels. This improves imaging quality. Rao et al. [24] enhanced the model’s robustness to noise. They added different degrees of artificial noise to the training set. Zhang et al. [25] introduced an attention mechanism into UNet. It guided the network to focus on damage-related regions. This suppressed background noise responses to a certain extent. The above studies also reveal two common shortcomings of existing methods. Single-direction scanning struggles to resolve the dependence of the response on the damage orientation, because one scan line observes the defect along a single direction. Additionally, there is a lack of effective anti-interference mechanisms for low-amplitude fluctuations. This leads to a high false detection rate.

To address the above bottlenecks, this paper proposes a CFRP damage detection method. It is based on a Dual-branch Artifact-Resistant Fast Imaging Network (DARF-Net). The method uses an air-coupled ultrasonic Lamb wave detection system. It performs line scans on CFRP plates along 0° and 90° directions. The signals from the two orthogonal directions are fed into two independent encoding branches. This fully extracts sensitive features for damage parallel and perpendicular to the fiber orientation. These features form a complement. The bottleneck layer uses element-wise addition fusion. It integrates dual-view information. This enhances true damage responses and suppresses single-view noise. Residual blocks are embedded in the decoder. They improve gradient propagation through identity mapping. This alleviates the gradient vanishing problem in deep networks. It improves convergence stability and reconstruction details. The network output undergoes morphological post-processing. This removes most spurious responses caused by material inhomogeneity or reconstruction errors. Thus, it effectively reduces the false detection rate. It improves detection precision and ensures the physical credibility of the imaging. This provides a feasible technical solution for field evaluation of CFRP structures.

## 2. Modal Analysis of Ultrasonic Guided Waves

Guided waves are excited and received in CFRP plates. The selection of modes directly affects detection sensitivity and signal-to-noise ratio. This paper aims to determine suitable guided wave modes and excitation parameters. First, the dispersion characteristics of guided waves in CFRP plates must be accurately solved. This solution is based on actual material parameters.

Frequency selection requires considering the center frequency response of the air-coupled transducer. It also requires considering the severe attenuation of high-frequency ultrasound in composite materials. A center frequency of 200 kHz is selected for air-coupled ultrasonic testing. This avoids a sharp signal-to-noise ratio (SNR) decrease. The decrease is caused by severe attenuation at higher frequencies. It also ensures sufficient spatial resolution to detect small damage. At this frequency, the A0 mode has a shorter wavelength. It provides higher sensitivity to small defects in the plate. Its dispersion is relatively flat near 200 kHz. This helps maintain the stability of the guided wave waveform.

**Figure 1:**
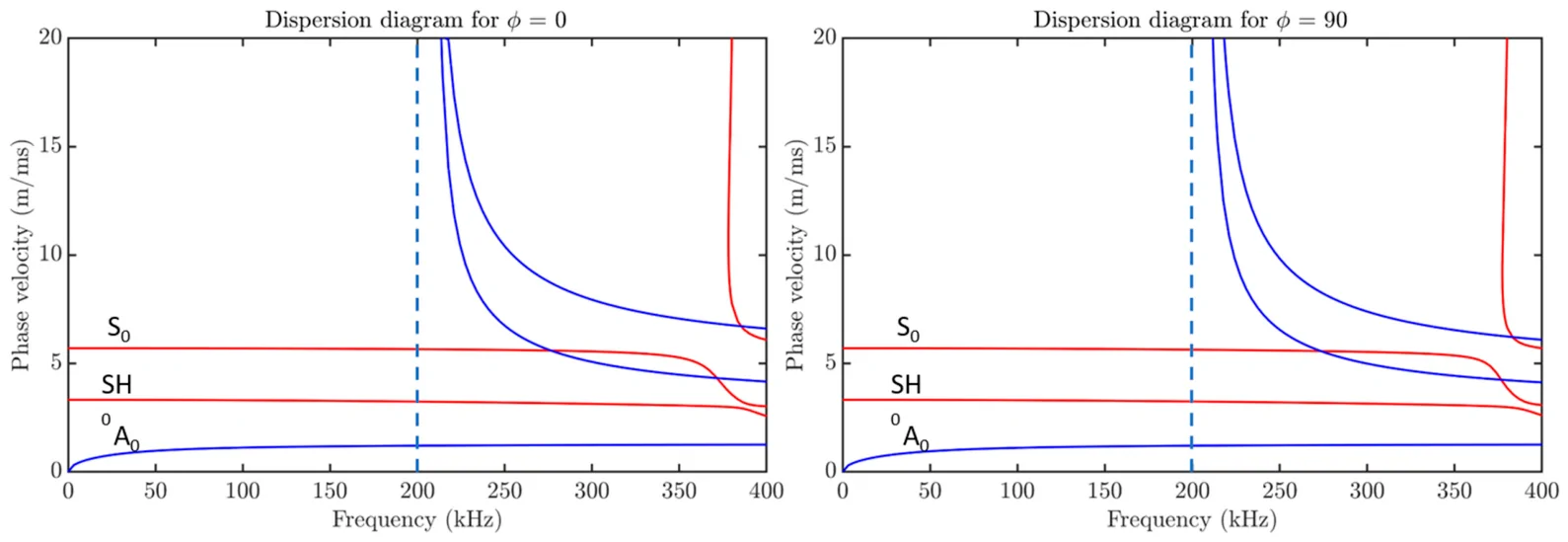
Dispersion curves along 0° and 90° directions.

This paper uses the engineering elastic constants and density of the CFRP plate listed in Table 1. It employs the numerical simulation software Dispersion Calculator. A semi-analytical method is adopted. This method calculates the dispersion characteristics and displacement field distribution of guided waves in the plate. Figure 1 shows the results. At a center frequency of 200 kHz, three fundamental guided wave modes mainly exist in the CFRP plate. These are the symmetric mode S_0_, the antisymmetric mode A_0_, and the shear horizontal mode SH_0_.

**Figure 2:**
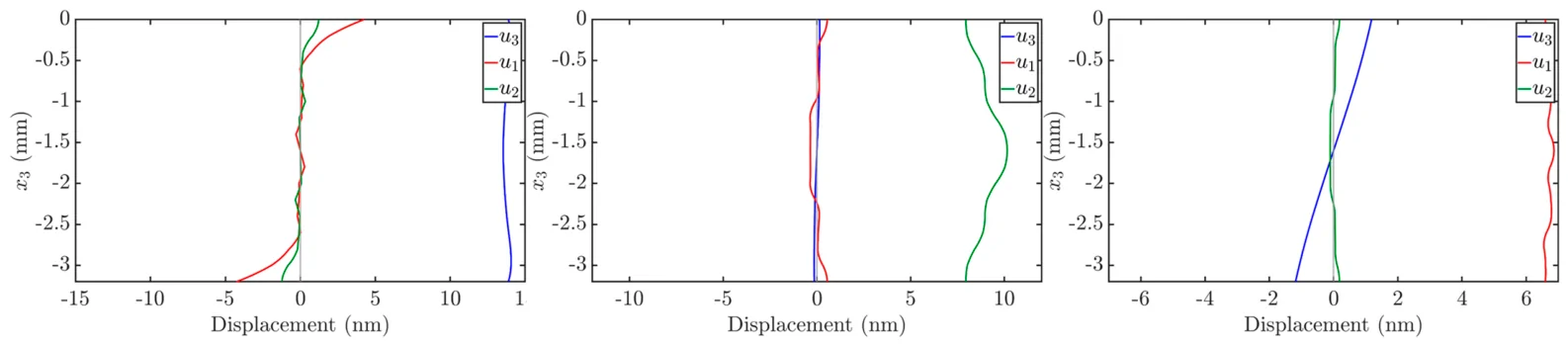
Displacement distribution characteristics along the plate thickness direction for A_0_, SH_0_, and S_0_ modes.

Figure 2 shows the displacement distribution characteristics along the plate thickness direction. These characteristics indicate that the A_0_ mode is dominated by the out-of-plane displacement component (u_3_) in the low-frequency range. This component is perpendicular to the plate surface. Its acoustic field energy easily leaks into the air medium outside the plate. This is highly beneficial for signal reception by air-coupled transducers. In contrast, the S_0_ mode is dominated by in-plane displacement (u_1_). The displacement of the SH_0_ mode is parallel to the plate surface (u_2_). Both struggle to effectively radiate acoustic waves into the air outside the plate. Their received signal strength is much weaker than that of the A_0_ mode. Therefore, considering the coupling efficiency, this paper selects the A_0_ mode as the preferred mode for air-coupled ultrasonic testing.

Effective excitation of guided wave modes requires strict adherence to the phase matching condition. This is also known as Snell’s law. When an ultrasonic beam is obliquely incident from air into the plate at a specific angle *θ*, the incident angle and the phase velocity of the mode in the plate satisfy the following relationship.

(1) $$\sin\;\theta =\frac{c_{\mathrm{air}}}{c_{p}}$$

Here, *c_air_* is the speed of sound in air (approximately 343 m/s). *c_p_* is the phase velocity of the excited guided wave mode in the plate. The CFRP plate has significant anisotropic characteristics. The phase velocity of guided waves differs along different fiber directions. This results in different optimal excitation angles for different scanning directions.

**Figure 3:**
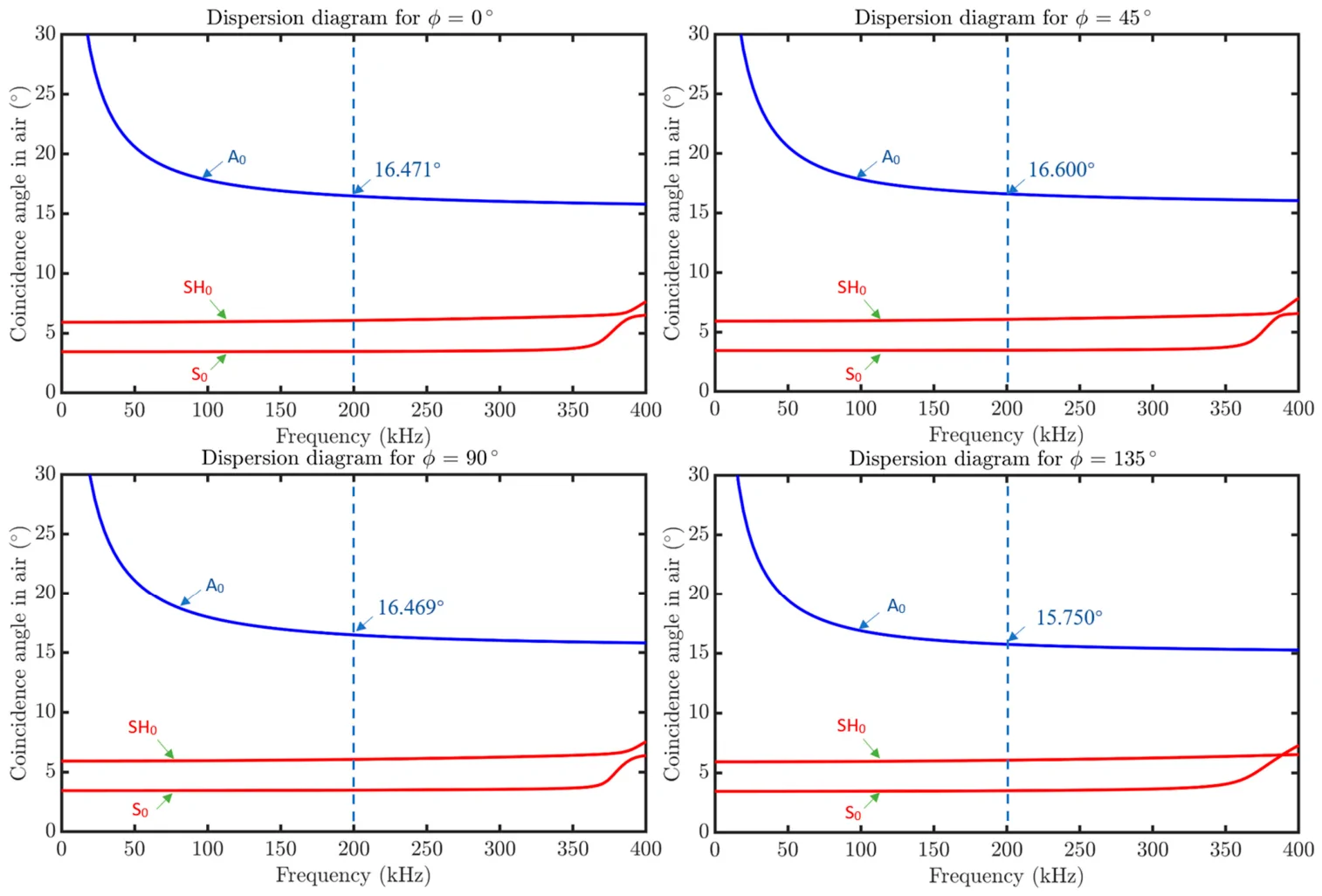
Angle selection for different ply orientations.

Figure 3 shows the coincidence angle of the A0 mode for four propagation directions (0°, 90°, 45° and 135°). At 200 kHz the optimal incidence angles of the four directions are 16.471°, 16.469°, 16.600° and 15.750°, respectively. The angular difference is not uniform: the 0° and 90° directions differ by only 0.002°, whereas the 45° and 135° directions differ by 0.850°, which is the largest difference among the directions examined. This direction dependence originates from the laminate: the plate is a balanced quasi-isotropic [−45/45/0/90] laminate whose in-plane stiffness matrix is isotropic, whereas the bending stiffness of the non-symmetric stacking sequence remains anisotropic, so that the angular spread reaches its maximum along the ±45° pair. Consequently, a single fixed incidence angle of 16.0° is appropriate for the 0°/90° configuration used here, since the phase-matching angles of these two directions differ by only 0.002°; the ±45° configuration would instead require a separate angle for each direction. Since the two scan directions are nearly equivalent in terms of wave propagation, the benefit of the dual-branch design is not related to material anisotropy: a single line scan provides only a one-dimensional projection of the damage position, whereas the two orthogonal scans provide two linearly independent projections of the same damage, which are jointly required for two-dimensional localization.

To ensure the consistency of the scanning system, this paper adopts a fixed incidence angle of 16.0°. This avoids experimental errors and operational inefficiency. These issues are caused by frequently changing the transducer angle in different scanning directions. It significantly simplifies the experimental procedure. It also improves the detection efficiency of omnidirectional scanning. Meanwhile, it ensures high excitation efficiency of the transducer.

## 3. Damage Imaging Method Based on Dual-Branch Artifact-Resistant Network

**Figure 4:**
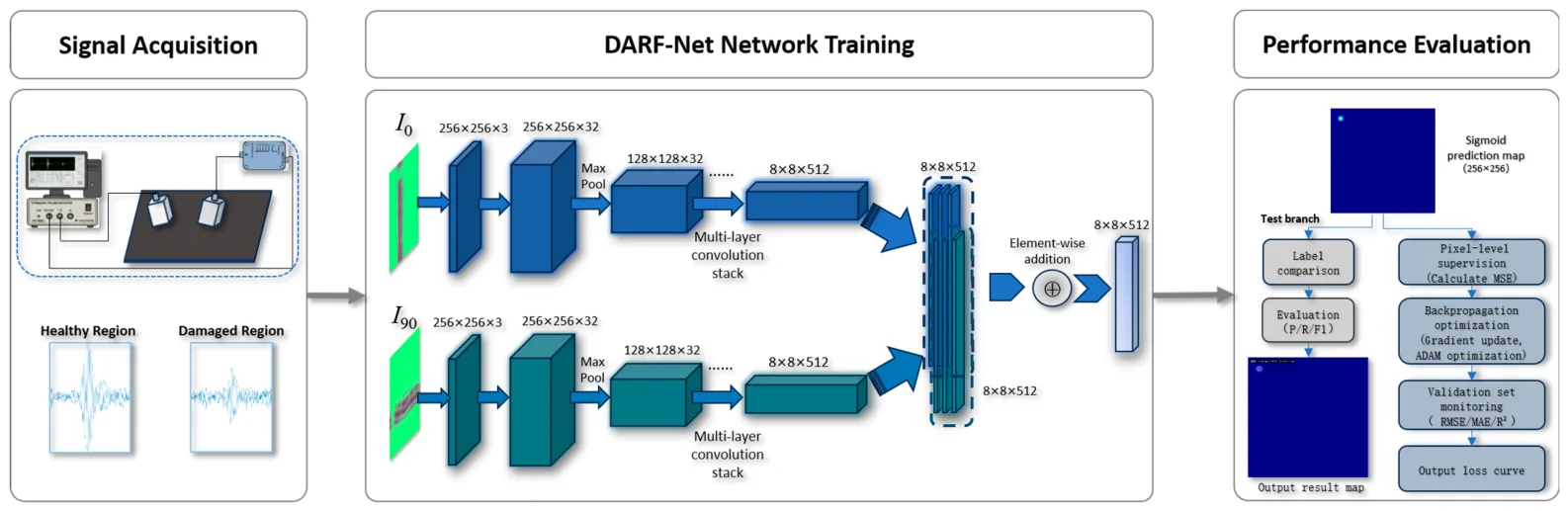
Flowchart of DARF-Net.

This chapter introduces a rapid damage imaging method. The method is based on the dual-branch artifact-resistant fast imaging network (DARF-Net). Figure 4 shows this method. It consists of three stages: signal acquisition, network training, and performance evaluation. These stages perform three tasks. The first is the acquisition of guided wave signals. The second is learning the relationship between guided wave features and real damage images. The third is evaluating the model’s performance in damage recognition.

### 3.1. DARF-Net Network Architecture

**Figure 5:**
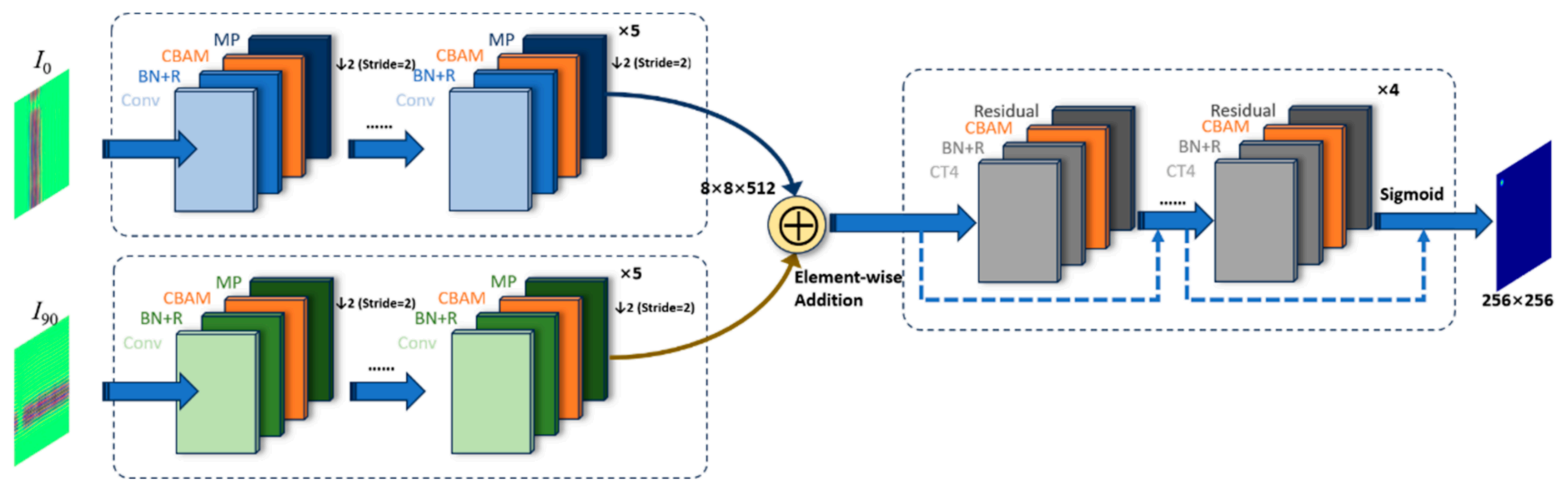
DARF-Net Network Architecture Diagram.

This paper designs the DARF-Net network. It targets orthogonal dual-view input and pixel-level image reconstruction. Figure 5 shows the overall architecture. The network consists of three components. The first is two encoder branches. They have identical structures but independent parameters. The second is a fusion layer. It uses element-wise addition. The third is a shared decoder with shared parameters. The core of this design is as follows. The two encoders extract specific feature information from the 0° and 90° views. The fusion layer integrates these complementary features. The decoder then reconstructs a high-fidelity damage probability distribution image. This reconstruction is based on the fused features.

Let the input images be *I*_0_ ∈ *ℝ*^3×256×256^ and *I*_90_ ∈ *ℝ*^3×256×256^. They correspond to the imaging results of data acquired in the 0° and 90° directions, respectively. Each encoder is composed of 5 stacked convolutional stages. Shallow stages extract primary visual features, such as edges and textures. Middle stages gradually abstract the contour and structural information of damage regions. Deep stages further encode the global context and the relative spatial relationship between the two views. Each stage contains two consecutive 3 × 3 convolutional layers (stride 1, padding 1). Batch normalization and ReLU activation follow them sequentially. A Convolutional Block Attention Module (CBAM) [26] is introduced at the end of each stage. Finally, a 2 × 2 max pooling is performed for spatial downsampling. CBAM sequentially performs channel attention and spatial attention weighting. This enables the network to adaptively emphasize damage-related channels and spatial regions. It suppresses interference from background noise and irrelevant textures. The number of channels increases gradually across stages: 32, 64, 128, 256, and 512. The spatial size is gradually reduced from 256 × 256 to 8 × 8. The output feature maps of the two encoding branches are denoted as

*F*_0_ = *E*_0_(*I*_0_), *F*_90_ = *E*_90_(*I*_90_) (2)

Here, *F*_0_ and *F*_90_ denote the bottleneck feature maps produced by the two encoders. The two encoders have identical structures but independent parameters, and each of them is applied to one of the two orthogonal scan directions. The fusion layer then combines the two feature maps by element-wise addition, *F* = *F*_0_ ⊕ *F*_90_. This fusion rule is adopted for two reasons. First, both branches are driven by the same physical quantity, namely the attenuation of the A_0_-mode amplitude along two orthogonal scan lines, and the two encoders are structurally identical, so their feature maps are expressed in a common, channel-aligned feature space. Under this condition, element-wise addition acts as feature-level ensembling: responses that are common to the two views, i.e., responses that originate from a genuine damage, are constructively reinforced, whereas responses that are specific to one scan direction, such as coupling drift, baseline wander and the low-amplitude tail aligned with the scanning path, are mutually uncorrelated between the two branches and are attenuated by the summation. Channel concatenation, in contrast, keeps the two views in disjoint channel subspaces: the decoder would have to decide, from limited training data, which subspace to rely on, and the direction-specific artifacts would remain available to it as independent evidence, which is exactly what the anti-artifact design aims to suppress. An attention-weighted fusion rule would require an additional gating sub-network whose parameters must be estimated from the same limited data, and the additional capacity is difficult to justify for two inputs that carry the same physical quantity. Second, element-wise addition preserves both the spatial size and the channel width of the single-branch feature map (512 × 8 × 8), so that the fused tensor can be fed directly into the decoder that was designed for a single branch; channel concatenation would double the bottleneck width and would require the first transposed-convolution layer to be re-designed, which increases the parameter count and the risk of overfitting. It should be noted that addition does not discard information: because the two encoders are kept independent, the two views are still extracted by different filters, and the summation is applied only after each view has been mapped into its own discriminative representation.

The decoder takes *D* and *F* as input. It progressively upsamples the features through five transposed convolution layers (ConvTranspose2d, kernel 4 × 4, stride 2, padding 1). This restores the spatial size from 8 × 8 to 256 × 256. Each transposed convolution layer is followed by batch normalization, ReLU activation, and a CBAM. This continuously strengthens damage-related spatial and channel features during reconstruction. Subsequently, residual blocks are embedded after the first four upsampling stages. These stages correspond to 256, 128, 64, and 32 channels. Their structure is defined as follows.

*x_l_*_+1_ = ReLU(*x_l_* + *F*(*x_l_*)) (3)

Here, *F* represents a stacked nonlinear transformation. This transformation consists of two 3 × 3 convolutional layers, batch normalization, and ReLU. In a deep decoding network, gradients must pass through multiple transposed convolutions and nonlinear activation layers during backpropagation. They are highly prone to decay or even vanish. Residual connections provide a shortcut path for gradients through identity mapping. This allows deep parameters to obtain effective updates. It significantly helps both training convergence speed and final reconstruction accuracy. The number of decoder channels decreases in reverse order: 256, 128, 64, 32, and 16. This symmetric design maintains the balance of the encoder–decoder structure. It also gradually reduces the computational cost of high-dimensional features. Finally, a 3 × 3 convolution compresses the channels to 3. A Sigmoid activation function then constrains the pixel-wise values to the [0,1] range. It outputs the predicted image $\hat{I}$. The Sigmoid function maps the network output to interpretable probability values here. A value of 0 represents no damage at that position. A value of 1 represents the highest damage probability. Intermediate values reflect the level of confidence.

In summary, the complete forward mapping can be uniformly expressed as follows.

(4) $$\overset{\wedge }{I}=D(E_{0}(I_{0})+E_{90}(I_{90}))$$

Mean Squared Error (MSE) is adopted as the loss function for network training. It measures the pixel-wise difference between the predicted image and the ground truth labels. Since the damage region occupies only a small fraction of the label image, the training task is a strongly imbalanced pixel-labelling problem, and the background dominates the value of the loss. A small MSE therefore indicates that the predicted image is close to the label image on average, but it does not by itself indicate that the damage region has been located. For this reason the pixel-level metrics are not used as the only criterion; they are complemented by a connected-component matching strategy that reports Precision, Recall and F1 together with the centre-positioning and diameter deviations of the matched damage regions, so that a missed small damage is penalised directly even when the global MSE remains low. MSE is retained as the training objective because it provides a smooth pixel-wise regression target for the probability map, while the class imbalance is handled at the evaluation level by the matching-based metrics.

(5) $$L=\frac{1}{N}\sum_{i=1}^{N}\left\|{\hat{I}}_{i}\right.-{\left.I_{\mathrm{gt},i}\right\|}_{2}^{2}$$

where *L* is the mean squared error loss value. *N* is the batch size. ${\hat{I}}_{i}$ is the predicted image of the *i*-th sample by the model. *I_gt_*_,*i*_ is the corresponding ground truth label image. $∥\cdot {∥}_{2}^{2}$ denotes the sum of squared pixel-wise differences. This loss function minimizes the pixel-wise difference between the predicted image and the label image. This drives the network to learn a nonlinear mapping. The mapping goes from dual-view inputs to the damage probability distribution.

The entire network adopts an end-to-end training strategy and requires no intermediate supervision. The only post-processing step is the morphological filtering applied to the network output at inference (Section 3.2); it contains no trainable parameters and therefore does not participate in training. The network takes dual-view images as input and directly outputs the reconstruction results. This greatly simplifies traditional multi-step cascaded imaging methods, reduces accumulated errors and removes the burden of parameter tuning. Since a single line scan resolves only the coordinate along the scanning line, the two orthogonal views must be used jointly by construction.

### 3.2. Morphological Post-Processing

**Figure 6:**
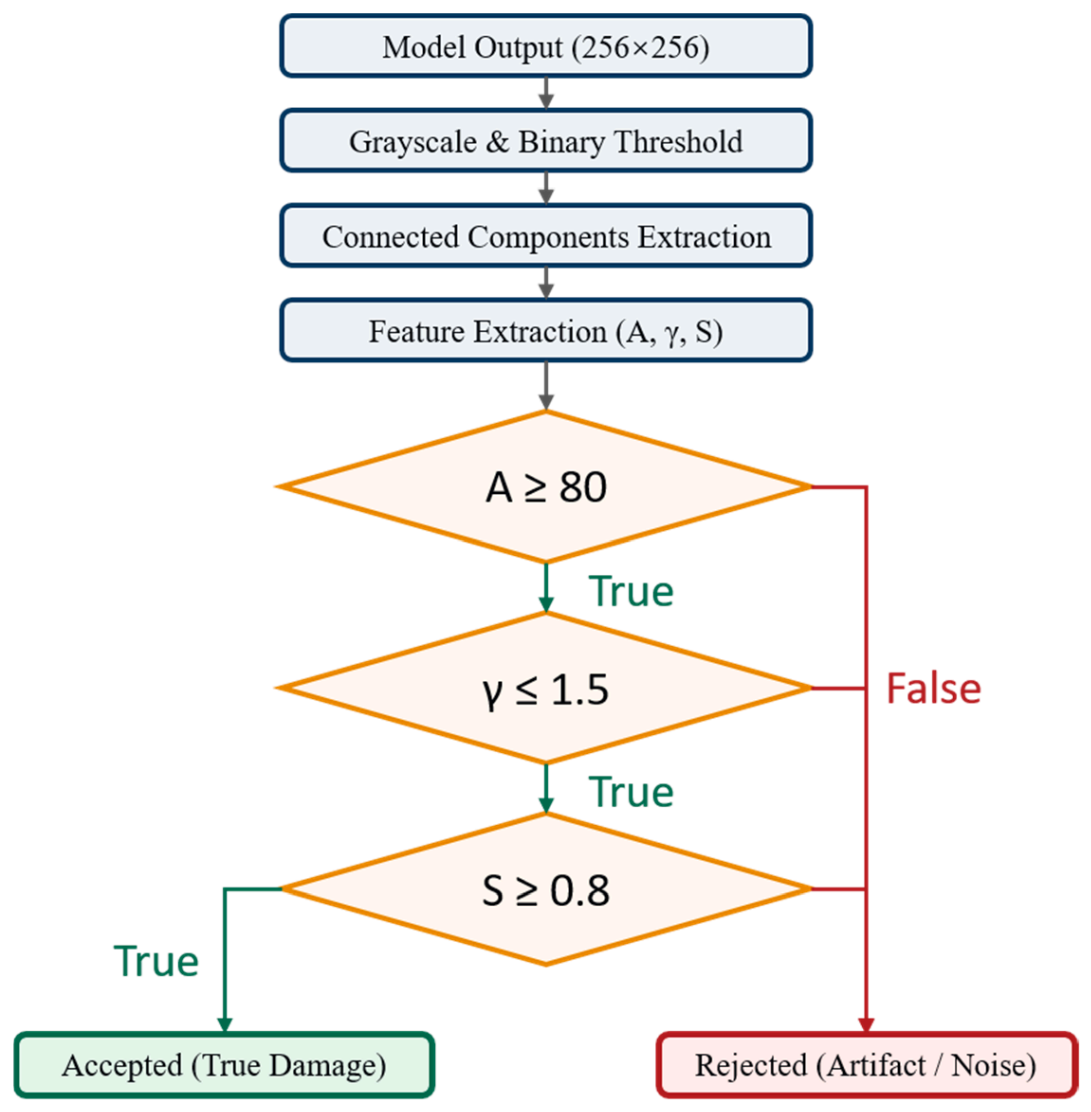
Flowchart of the filtering process.

To further remove residual boundary artifacts, this paper introduces morphological post-processing during the inference stage (Figure 6). It extracts true damage regions from the model output probability map through geometric feature screening. The raw output image of the model is a three-channel RGB probability map. Each channel encodes the spatial distribution of damage probability. First, the RGB image is converted into a single-channel grayscale image. The grayscale values range continuously from 0 to 1. They reflect the model’s confidence that each pixel belongs to a damage region. Then, the grayscale image is binarized according to a preset threshold τ. Pixels with grayscale values greater than τ are marked as candidate damage regions. Pixels with values less than τ are treated as background and set to zero. This operation converts the continuous probability map into a binary mask. It preliminarily distinguishes potential damage from background regions.

After binarization, all connected components are extracted. Three geometric feature parameters are calculated for each connected component. These parameters are pixel area A, aspect ratio γ, and solidity S. Pixel area A is the total number of pixels within the connected component. It is used to constrain the lower limit of the damage scale. The threshold A ≥ 80 is based on physical mapping. The physical sample size is 100 mm × 100 mm. This corresponds to a resolution of 256 × 256 pixels. Based on this, a circular damage with a diameter of 5 mm should theoretically occupy about 128 pixels in the image. During binarization, edge pixels may shrink slightly due to threshold selection. To avoid mistakenly filtering out true micro-damage, the area threshold is lowered to 80 pixels as a safety margin. The equivalent circular diameter is about 4 mm. This is slightly lower than the minimum physical size of real damage, which is 5 mm. This ensures that all true damage can pass the area screening.

The aspect ratio γ is defined as the ratio of the long side to the short side. This refers to the minimum bounding rectangle of a connected component. It characterizes the shape anisotropy of the damage region. True damage usually appears nearly circular or elliptical in the plane. Its aspect ratio generally does not exceed 1.5. In contrast, artifacts often appear as elongated strip-like shapes. These artifacts include signal tails and boundary reflections in the scanning direction. Their aspect ratios are significantly larger. Setting γ ≤ 1.5 can effectively filter out these strip-like artifacts.

Solidity S is defined as the ratio of the pixel area of a connected component to its convex hull area. It is a metric for measuring the compactness of region filling. True damage regions consist of dense damage response pixels. The difference between their convex hull area and pixel area is small. Thus, their solidity is close to 1. In contrast, spurious responses caused by noise or reconstruction errors usually show loose and irregular distributions. Their pixel areas are much smaller than their convex hull areas. Thus, their solidity is significantly low. A lower limit of S ≥ 0.8 is set. This ensures the retention of densely filled true damage. Meanwhile, it excludes scattered noise point clusters from the candidate regions.

The three geometric parameters are applied in sequence. The order is area, aspect ratio, and solidity. Only connected components that simultaneously satisfy A ≥ 80, γ ≤ 1.5, and S ≥ 0.8 are classified as true damage. All other regions are removed as artifacts. This joint screening strategy accounts for three factors. They are the physical plausibility of damage size, the compactness of shape, and the filling integrity of the region. It maintains high recall. It also effectively controls the false detection rate. As a result, the final imaging result better matches the physical priors of damage in spatial distribution.

## 4. Experiments

### 4.1. Experimental Setup

All experiments are conducted on a laptop. The laptop runs Windows 11. Its CPU is an Intel Ultra9-275HX. Its GPU is an NVIDIA RTX5070. It has 32 GB of RAM. The Python version is 3.14. The PyCharm version is 2026.1. The optimizer is Adam. The initial learning rate is set to 10^−3^. The weight decay coefficient is 10^−5^. An adaptive learning rate scheduling strategy is introduced during training. The learning rate is halved when the validation loss does not decrease for 5 consecutive epochs. The batch size is set to 8. The total number of epochs is 60. After each epoch, the validation loss is calculated. The current best model weights are saved.

**Figure 7:**
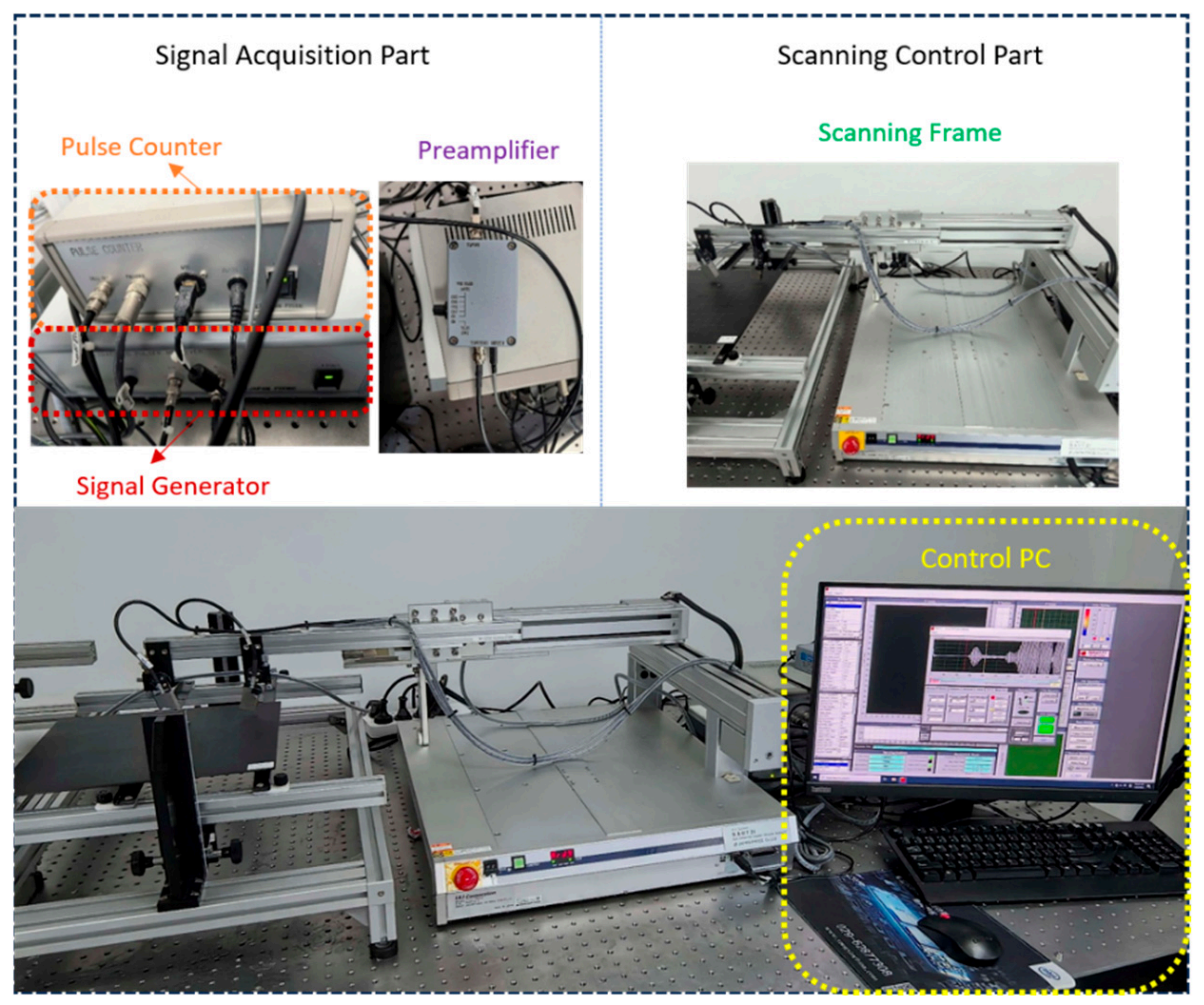
Air-coupled ultrasonic experimental system.

The experiment uses a CFRP damage detection system. This system is based on an air-coupled ultrasonic setup. Figure 7 shows the structure of the experimental system. The experimental equipment is a high-sensitivity non-contact air-coupled ultrasonic testing system. It is manufactured by Japan Probe Co., Ltd in Yokohama, Japan. Its model is NAUT21-1. Its software is developed based on LabVIEW. The scanning system consists of control software, a signal generator JPR600C, a signal acquisition device, a preamplifier, a computer, and a two-dimensional scanning frame. The air-coupled ultrasonic transducer has a size of 14 mm × 20 mm. Its center frequency is 0.2 MHz. The detection system outputs a rectangular excitation signal through the signal generator JPR600C. The pulse voltage is set to 220 V. The center frequency is matched with the transducer. It is set to 0.2 MHz. The high-pass and low-pass filters are set to 0.1 MHz and 0.3 MHz, respectively. The received signal is amplified by a preamplifier with a gain of 60 dB. It is then acquired by the signal acquisition device at a sampling frequency of 20 MHz.

### 4.2. Dataset Construction

**Figure 8:**
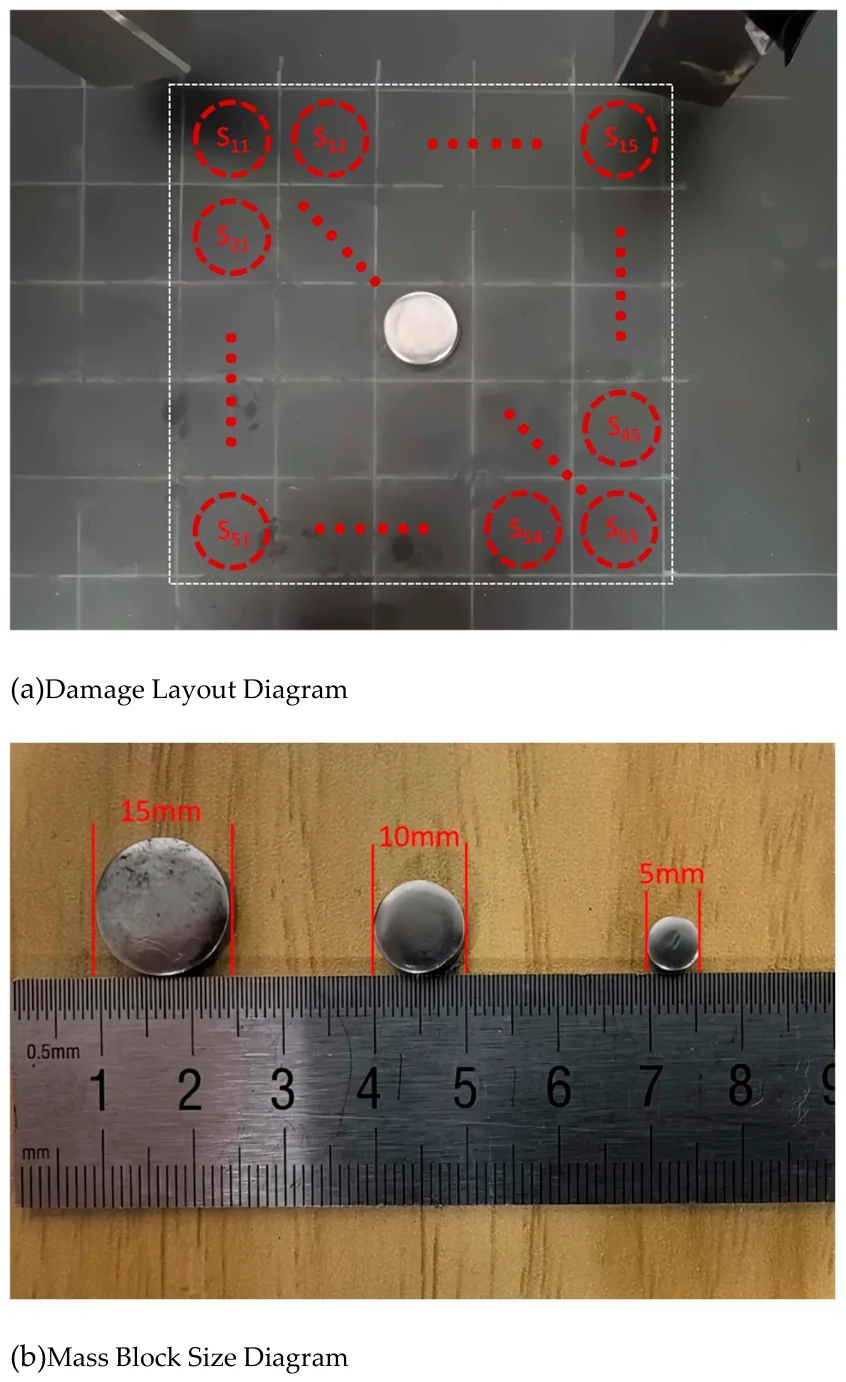
Damage layout and schematic diagram.

This paper uses a cross-scanning mode to acquire the dataset. The air-coupled ultrasonic transducers are arranged in a single-transmitter single-receiver configuration. This effectively alleviates the signal imbalance problem in traditional piezoelectric sensor arrays. Two T700 carbon fiber composite plates are used in the experiment. Their dimensions are 300 mm × 300 mm × 3.2 mm. The carbon fiber plies are stacked alternately in the order of −45°, 45°, 0°, and 90°. Each plate is divided into four 100 mm × 100 mm regions. Each region is further subdivided into 25 grids of 20 mm × 20 mm, as shown in Figure 8a. During the experiment, mass blocks with diameters of 5 mm, 10 mm, and 15 mm are fixed sequentially in these grids using a couplant, as shown in Figure 8b. This simulates damage of different sizes. Previous studies have shown that fixing mass blocks on composite plates with a couplant can produce guided wave changes similar to actual damage [27,28]. Before data acquisition, a 100 mm × 100 mm region on the CFRP plate is selected. Line scans are performed along the 0° and 90° directions. The step size is set to 1 mm. This region generates a total of 202 healthy signals. These signals serve as baseline reference signals. Then, mass blocks are attached to each grid. Each dataset group contains a total of 202 signals from both the 0° and 90° directions. The composite plate has anisotropic properties. Thus, the guided wave signal energy differs slightly between the two scanning directions. Before each acquisition, the transducer is placed in a healthy region. The gain is adjusted to keep the signal amplitude in both directions at approximately 0.7 V. This ensures consistency of the control variables.

Coupled mass blocks are used as the damage model for four reasons. First, real low-velocity impact damage is destructive and irreversible: producing one impact permanently consumes the specimen, because a delamination cannot be removed or repositioned, so covering the three nominal sizes, the 25 grid positions and the two scanning directions would require a very large number of plates. Second, the size of an impact delamination cannot be prescribed quantitatively: its lateral extent and through-thickness morphology are governed by the impact energy, the boundary conditions and the stochastic fibre–matrix interaction, so that the nominal classes of 5 mm, 10 mm and 15 mm on which the size-accuracy evaluation relies cannot be realised in a controlled way. Third, the damage position cannot be adjusted: moving a defect to another grid position would require producing a new impact. Fourth, changing the position by moving the scanning frame is error-prone, because the translation stage must be re-zeroed and re-aligned for every change in position, and the resulting repositioning uncertainty would be confounded with the localization error that this work aims to quantify. Coupled mass blocks avoid all four problems: the diameter is prescribed exactly, the block can be moved between the 25 grid positions without destroying the plate, the healthy baseline of the same specimen remains available, and the position is set by the scanning frame alone. It is nevertheless emphasized that the surrogate is not asserted to be equivalent to a real delamination: a coupled mass block modifies the local impedance and the wave path, but it does not reproduce the through-thickness morphology of a delamination nor the multiple scattering inside the delaminated interface. The quantitative accuracies reported in this work therefore characterise surrogate damage, and validation on real low-velocity impact damage with an independent reference measurement is identified as the first item of future work. The data from one plate constitute the training set. The data from the other plate constitute the test set. This fundamentally avoids data leakage.

**Figure 9:**
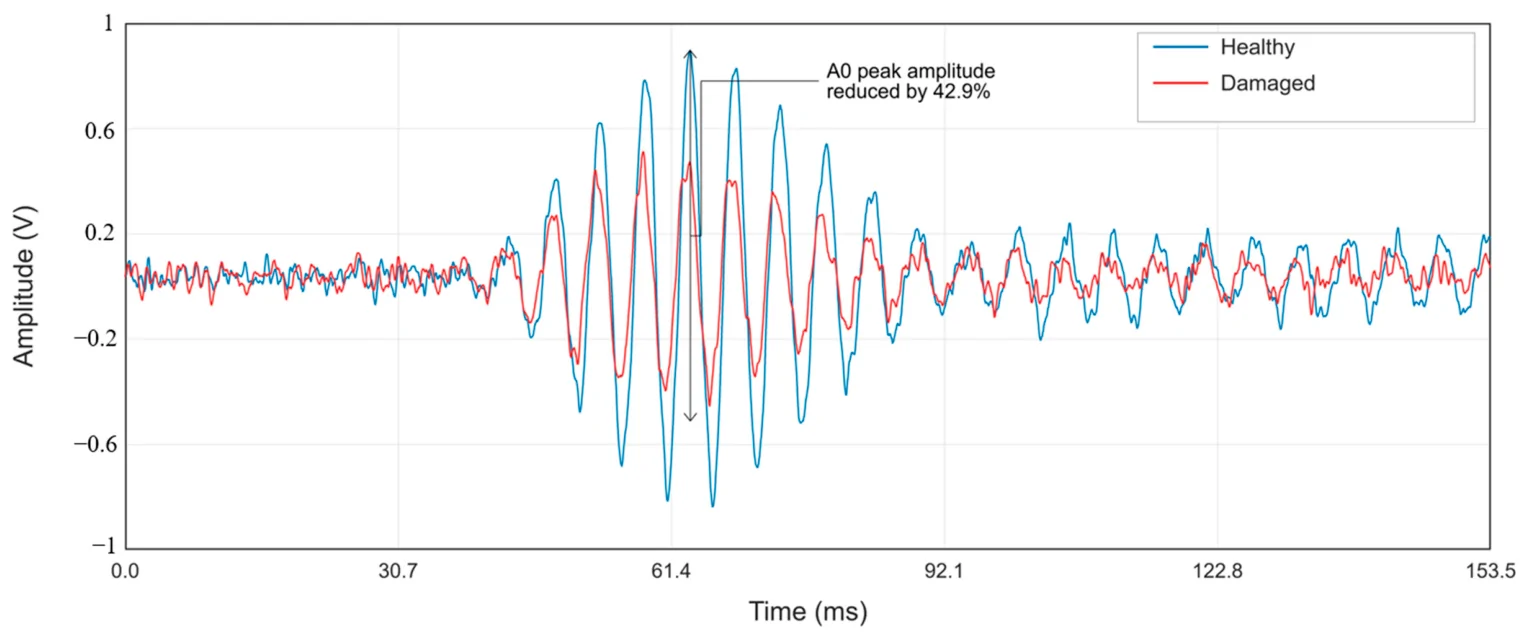
Time-domain A_0_ waveforms measured on the same scan line.

**Figure 10:**
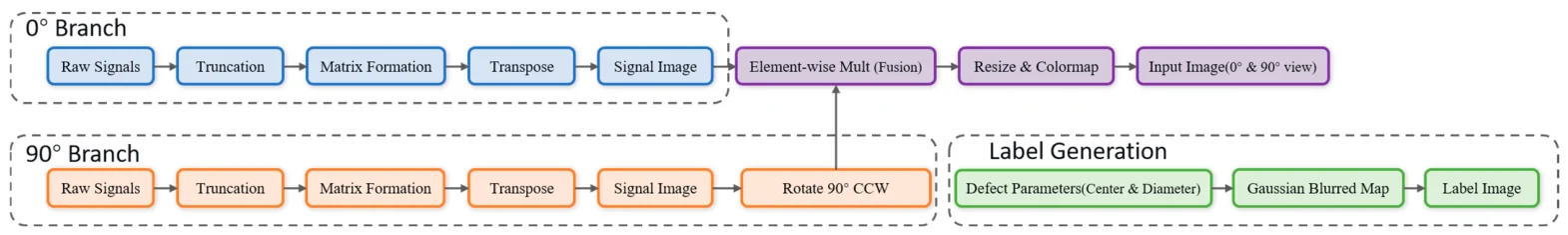
Flowchart of data preprocessing and label generation.

Time-domain waveforms are obtained at each scanning position. For comparison, Figure 9 shows the signals acquired at a healthy measurement point and at a damaged measurement point of the same scan line. The peak amplitude of the A_0_ wave packet of the damaged signal is 42.9% lower than that of the healthy signal, so that the scattering and attenuation caused by the damage are directly visible in the time domain. This is the physical basis on which the network performs damage localization. The middle effective interval is first intercepted. This focuses on damage-related signal components. The intercepted signals from 101 measurement points along the scanning path are arranged into a matrix. This arrangement follows the spatial order. Each row represents the intercepted waveform at one measurement point. The row direction corresponds to the spatial scanning position. The column direction corresponds to the time sampling points. The matrix is then rendered as a two-dimensional image, which constitutes the direct input of the network: the extracted waveform of each measurement point is one row of the image, so that the vertical axis of the image is the scanning position and the horizontal axis is the time sample within the intercepted interval. The matrix is 101 × 3070 before rendering and is resized to 256 × 256 pixels, and the amplitude of each sample is mapped to colour through the jet colour map, so that the three colour channels of the resulting image encode the same scalar amplitude. The network therefore receives a 3 × 256 × 256 tensor in which the three channels are the colour representation of the signal amplitude rather than three independent physical channels. One sample consists of the 0° image and the 90° image of the same measurement group, which are fed to the two encoder branches as two separate 3 × 256 × 256 inputs. The complete procedure of data preprocessing and label generation is summarized in Figure 10. Healthy regions have high signal amplitudes and a uniform distribution. They appear as a dark high-value band across the middle of the image. When Lamb waves encounter damage, scattering and attenuation occur. These effects reduce the local signal amplitude. This reduction appears as a low-value depression within the high-value band. It intuitively indicates the spatial location of the damage. This signal matrix is itself a feature image. It contains both spatial and temporal information. It can be directly used as network input for damage identification. Two data augmentation operations are applied to the original time-domain signals. (i) Gaussian white noise with a standard deviation uniformly sampled from 1% to 10% of the standard deviation of the corresponding signal is added to every channel. (ii) Random channel corruption: 10 to 20 of the 101 channels (9.9% to 19.8%) are selected at random and their amplitudes are attenuated by a factor uniformly sampled from 0.6 to 0.8, with an additional noise term of the same 1% to 10% level. Each measurement group is expanded into eight samples, namely one original, two noise-injected and five channel-corrupted samples, and 16 measurement groups are acquired for each nominal damage size. A total of 2400 samples are collected. The training and validation sets are drawn from one CFRP plate in a ratio of 8:2 with a fixed random seed, while the second CFRP plate is reserved exclusively for testing. The partition is performed at the level of the measurement group rather than the individual sample, so that the eight augmented versions of one acquisition always remain in the same subset, and the two plates share no identical image content.

### 4.3. Experimental Results and Discussion

#### 4.3.1. Model Training

**Figure 11:**
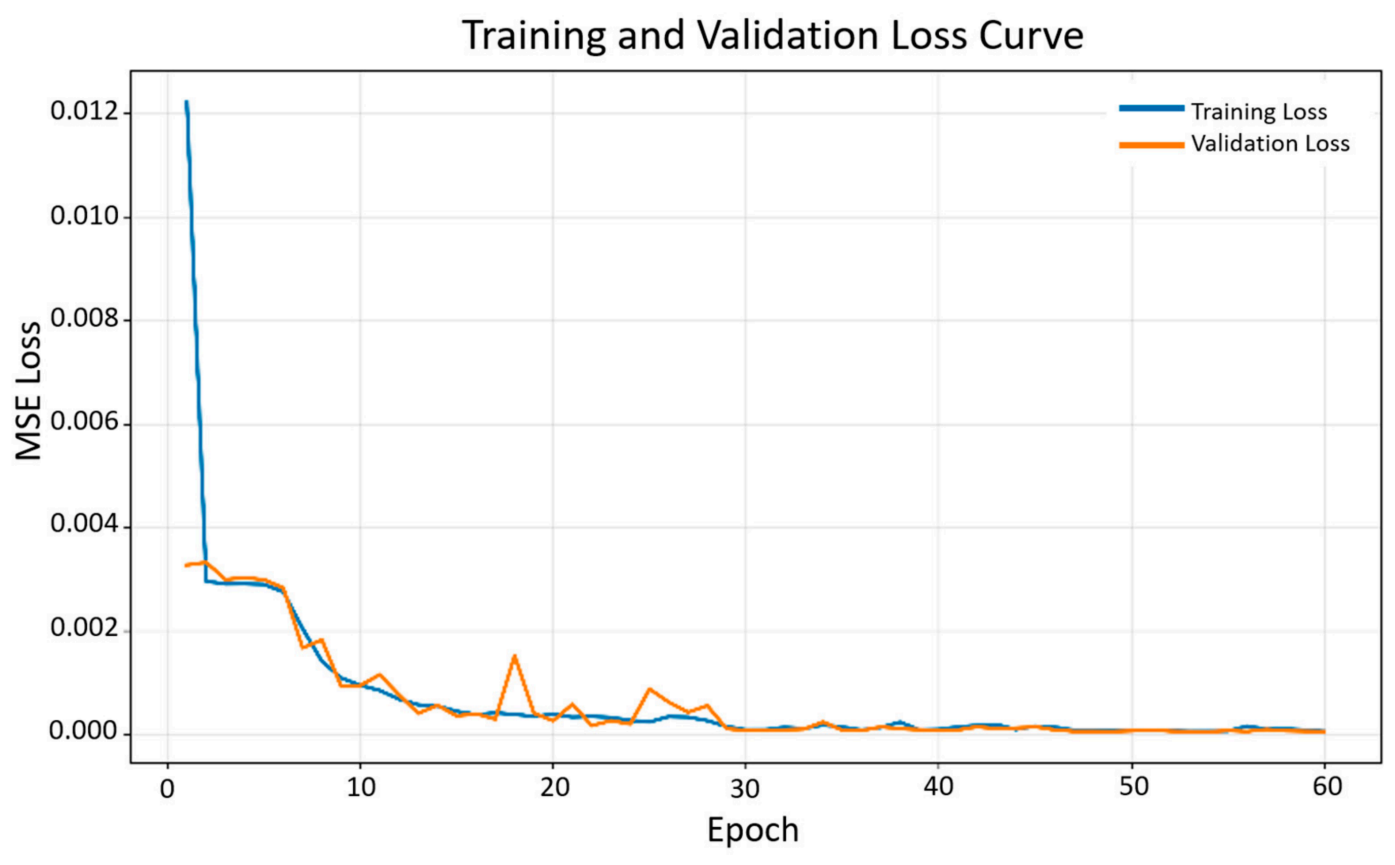
Training and validation loss curves.

The model is trained on a dataset containing 2400 samples. The dataset is divided into a training set and a validation set at a ratio of 8:2. The training and validation loss curves recorded during training are shown in Figure 11.

The loss curves show the following. As the number of training epochs increases, both training and validation MSE losses decrease rapidly and then stabilize. The two curves remain well synchronized. No obvious overfitting occurs. This indicates that the model has good generalization ability.

To comprehensively evaluate imaging quality, this paper adopts a multi-dimensional evaluation strategy. It introduces mean squared error (MSE), root mean square error (RMSE), mean absolute error (MAE), and the coefficient of determination (R^2^). These metrics evaluate the reconstruction quality of the model. They also evaluate its damage discrimination capability.

Regarding the morphological recovery capability of damage regions, the damage distribution is a key concern in practical applications. This paper introduces an evaluation strategy based on connected component matching. The post-processed predicted binary map is matched with the ground truth binary map component by component. The Intersection over Union (IoU) criterion is adopted. For each predicted connected component, a condition is checked. If its pixel overlap with a ground truth component exceeds a threshold θ, and this ground truth has not been matched yet, it is counted as a true positive (TP). Otherwise, it is counted as a false positive (FP). Unmatched ground truth components are counted as false negatives (FNs). Based on this, the precision, recall and F1-score are calculated. This set of metrics finely reflects the model’s recovery of damage location, size, and shape from a morphological perspective. It compensates for the insufficient sensitivity of pixel-level global metrics to local structures. After training, the detailed evaluation metrics on the validation set are listed in Table 2.

It should be noted that the morphological post-processing is not part of the learned model but a deterministic, parameter-free inference stage, and that it is applied in exactly the same way to the predicted probability map of every model compared in this work. The training objective therefore remains the pixel-wise reconstruction of the damage probability map, and the post-processing does not introduce any change in the optimisation target: it only converts the continuous probability map into a binary map and removes candidate regions whose geometric properties contradict the physical priors of damage. Because the module contains no trainable parameters and is applied identically at test time for all configurations, it cannot bias the comparison between the models. A quantitative check is provided by the ablation study: the network is trained once with the MSE objective and the same weights are then evaluated both with and without the post-processing, and the two results differ in precision and in F1 but not in the reconstruction of the damage probability distribution itself, which confirms that the post-processing acts on the decision level rather than on the training objective.

Table 2 shows the following. After 60 epochs of training, the validation MSE is only 5.5 × 10^−5^. This indicates that the model has a significant suppression capability for noise and artifacts in ultrasonic images. The extremely low MAE further demonstrates that the predicted image and the ground truth have a highly consistent pixel-level distribution across the entire image. The R^2^ value is close to 1. This indicates that the model has a strong ability to distinguish between damage regions and background. It can effectively preserve the spatial dynamic range and edge details of damage morphology. These metrics jointly verify that the model has good reconstruction accuracy and generalization performance after sufficient training. It can provide reliable prediction results for subsequent testing tasks.

#### 4.3.2. Model Testing and Damage Imaging Analysis

The trained model is used for systematic evaluation on the test set. The test set contains 200 samples. All samples are randomly selected from the data of a second CFRP plate of the same material. Regarding the morphological recovery capability of damage regions, a connected component matching strategy is adopted. It performs component-by-component pairing statistics between the predicted binary map and the ground truth binary map. For the 200 test samples, Precision, Recall, and F1 reach 0.9899, 0.9800, and 0.9849, respectively. These results show that most true damage is effectively identified. Meanwhile, the post-processing filtering mechanism successfully suppresses the vast majority of isolated artifacts. These artifacts are caused by noise or reconstruction errors. Both precision and recall exceed 0.9800. This fully demonstrates the high accuracy and reliability of the proposed method in air-coupled ultrasonic damage imaging tasks.

**Figure 12:**
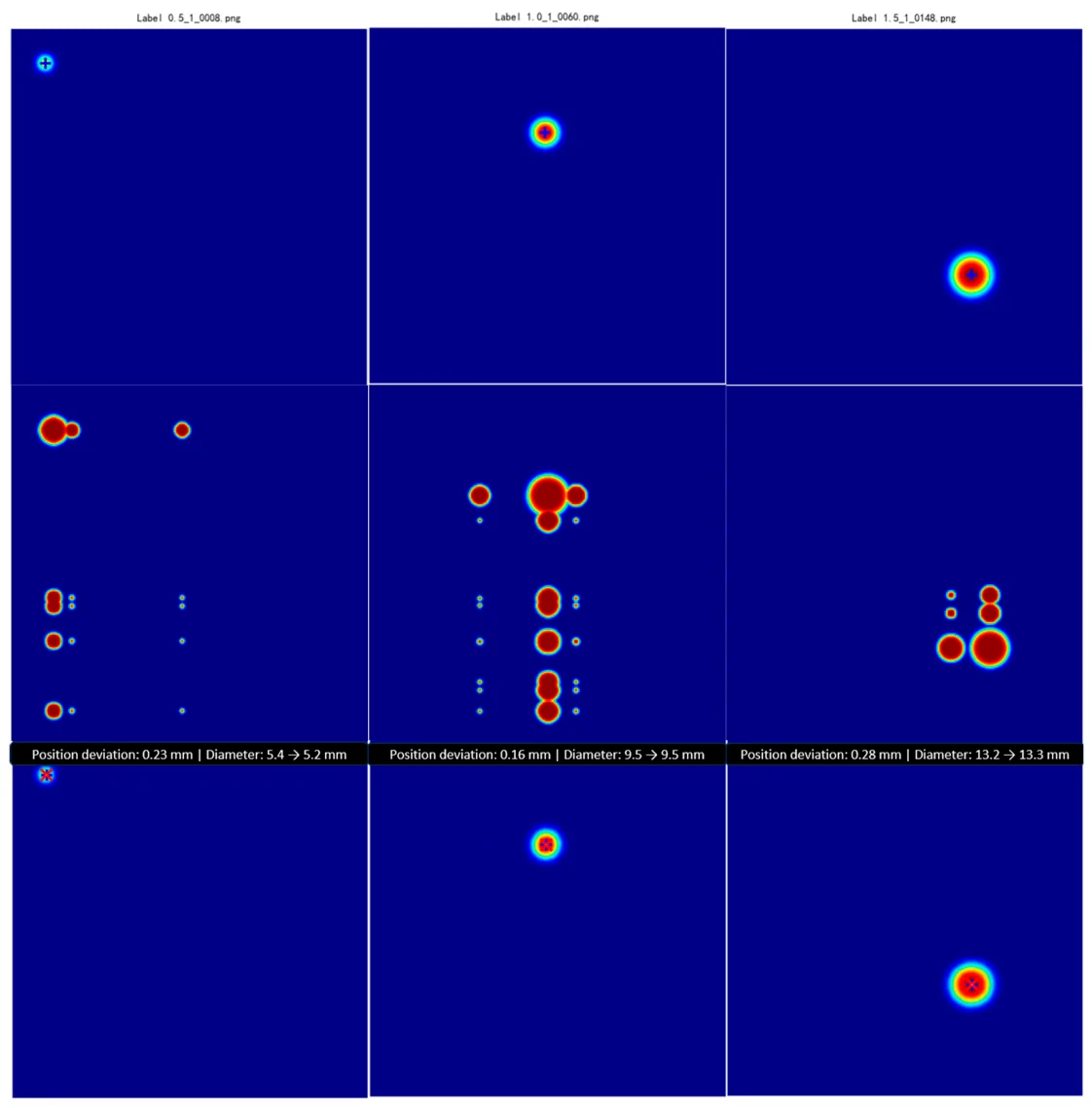
Comparison of damage information.

To comprehensively evaluate the overall performance of DARF-Net, this paper analyzes the test set results from two levels. These levels are visual presentation and quantitative statistics. The performance includes damage morphology recovery, spatial localization, and size estimation. Figure 12 randomly selects typical samples of three sizes from the test set. These sizes are 5 mm, 10 mm, and 15 mm. It shows a comparison of three rows. The first row is the ground truth images. The second row is the damage images directly generated by simple cross-localization. This method uses only the 0° and 90° images. The third row is the DARF-Net prediction images. The simple direct imaging method lacks the ability to discriminate noise and artifacts. It generates many spurious damage responses in the images. It cannot accurately restore the location and morphology of true damage. In contrast, the damage regions output by DARF-Net highly match the ground truth in location, shape, and size. Their edge contours are clear. There is no significant noise residue in the background regions. Even at non-ideal positions such as scanning edges, the prediction results can still accurately locate the damage center. They can also completely restore the damage morphology. This comparison intuitively verifies the significant advantages of deep learning methods. These advantages lie in damage feature extraction and anti-artifact capability. They are superior to traditional direct imaging methods.

In addition to the well-performing samples above, the test set also contains a few samples with typical errors. Figure 12 shows typical samples with deviations in three dimensions. These dimensions are size estimation, connected component matching, and center positioning. For the size estimation error sample, the predicted damage diameter is about 0.700 mm larger than the ground truth. In the connected component matching error sample, the model produces a weak isolated response before post-processing. This response is marked by a red circle. It is finally falsely detected as damage. For the center positioning error sample, the predicted center deviates from the ground truth center by about 1.600 mm. This corresponds to an offset of about 4 pixels. These errors account for a very low proportion in the overall test set. Their deviation magnitudes are all within an acceptable range. They do not affect the overall evaluation conclusion. However, they help us intuitively understand the performance differences in the model on various samples.

**Figure 13:**
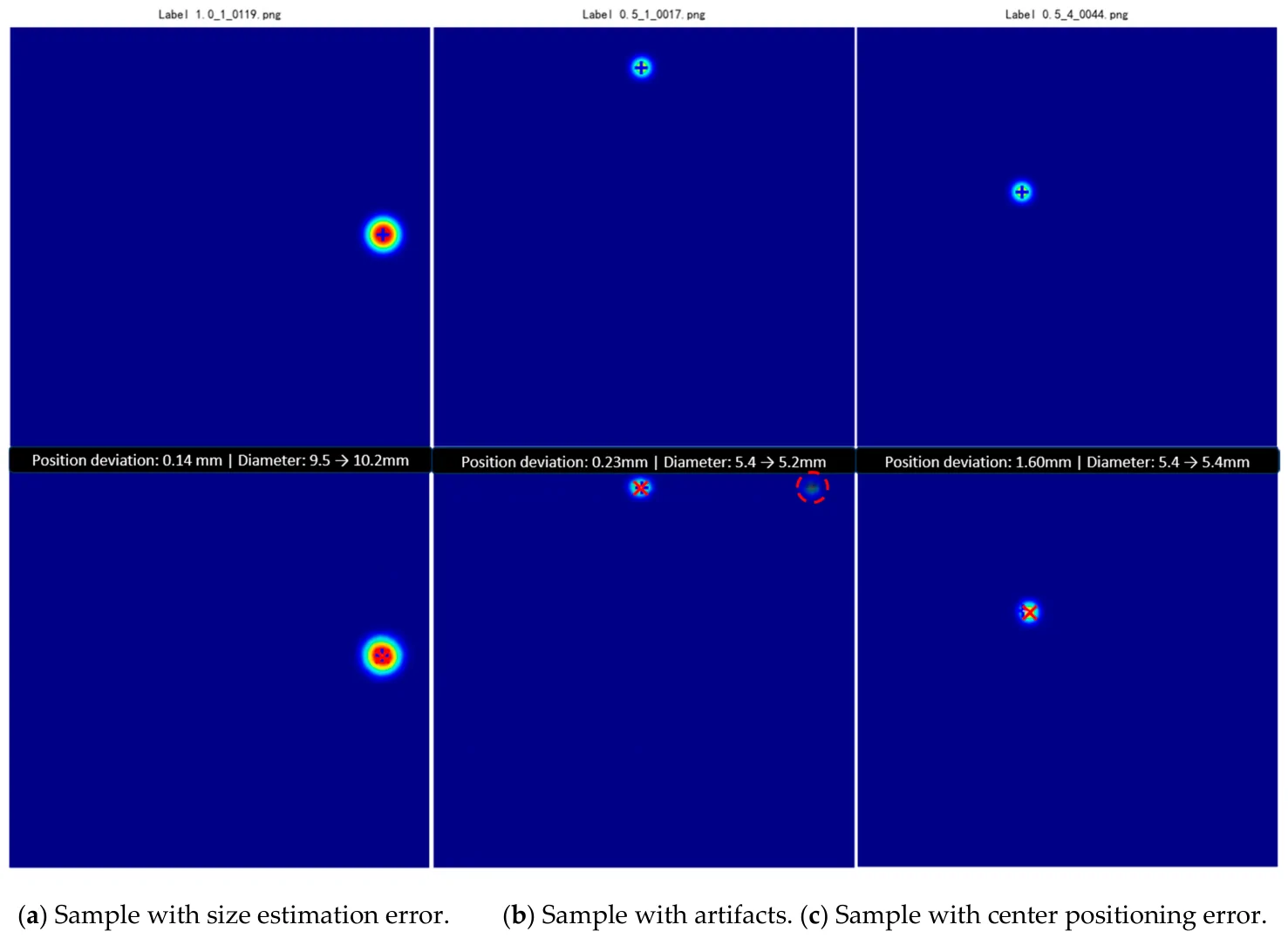
Prediction results of three typical error samples in the test set.

Regarding spatial localization accuracy, Table 3 shows the statistical results of the 200 test samples. The mean deviation between the predicted damage center and the ground truth center is 0.133 mm. The standard deviation is 0.247 mm. The maximum deviation is 1.194 mm. The damage diameter ranges from 5 to 15 mm. This deviation level has little impact on actual detection results. In Figure 13, blue “+” and red “×” mark the ground truth centers and predicted centers, respectively. This intuitively shows the visual representation of the localization deviation.

Regarding size estimation accuracy, statistics are calculated for the diameter deviations of 200 matched connected component pairs (Table 4). The mean absolute deviation between the predicted diameter and the ground truth diameter is 0.188 mm. The standard deviation is 0.501 mm. The maximum deviation is 1.875 mm. This deviation magnitude accounts for a very small proportion of the damage size. This indicates that the model also has good accuracy in the quantitative estimation of damage size. It can provide a reliable basis for damage severity assessment in practical engineering. In Figure 13, the top of each predicted image is annotated with size change information. The format is “ground truth diameter → predicted diameter”. This allows an intuitive comparison of their size consistency.

Integrating the above visual and quantitative results, this paper proposes a dual-branch reconstruction network. This network is combined with a post-processing filtering strategy. It achieves high accuracy in three dimensions. These dimensions are damage morphology recovery, center positioning, and size estimation. This validates the effectiveness of dual-view feature fusion and residual decoding structures. These structures are applied in air-coupled ultrasonic damage imaging.

#### 4.3.3. Generalization Validation

The test set in the above imaging analysis comes from 25 discrete grid positions. The model shows excellent performance on this set. This demonstrates its good fitting capability. However, in practical inspection the damage position is not restricted to the training grid. The localization accuracy of the model for unseen positions is critical. This is key to measuring its engineering practicality. Therefore, this paper selects 16 grid intersection points as new test points. These points are offset by 10 mm from the training grid centers. They are distributed at region centers and edges. Mass blocks with diameters of 5 mm, 10 mm, and 15 mm are attached at each position. This simulates damage. Signals are acquired following the same procedure. Input images are then generated. The trained model performs direct inference. The same morphological post-processing is applied.

**Figure 14:**
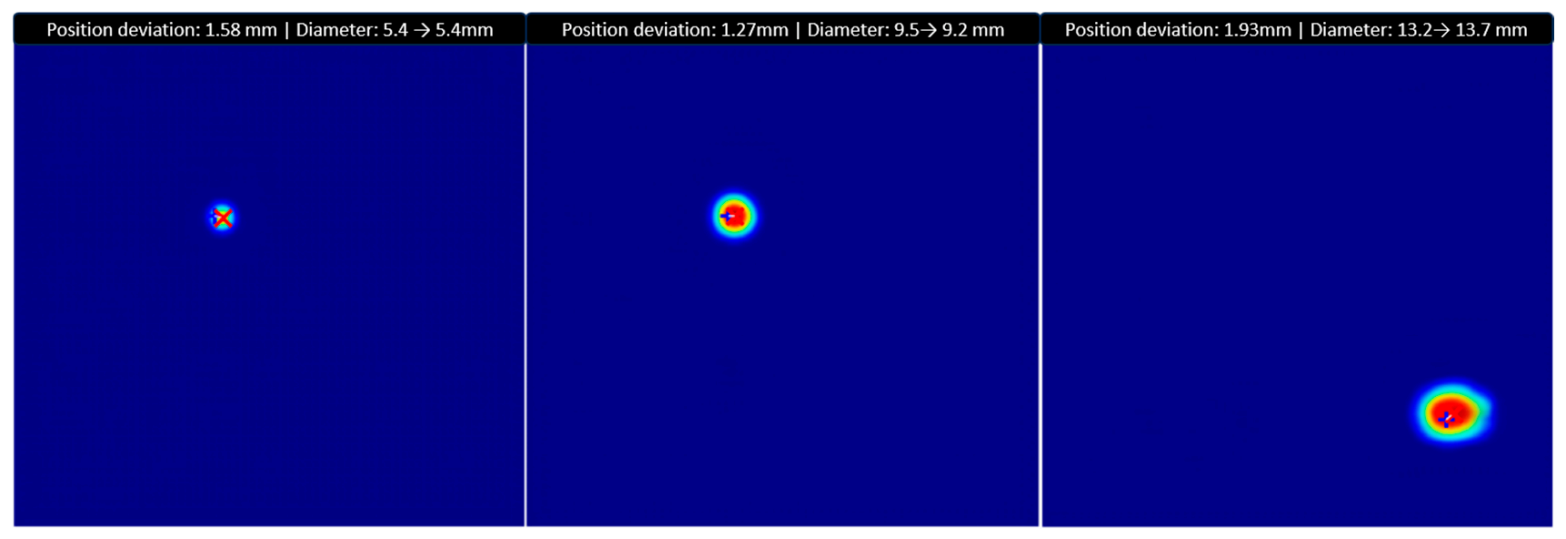
Comparison of damage information at non-training positions.

Figure 14 shows a comparison of the ground truth and the prediction for three representative samples at intersection positions. The damage regions output by the model remain highly consistent with the ground truth in location, shape and size, and the background shows no significant artifacts. To quantify the generalisation beyond the training grid, the complete statistics over all 16 intersection positions and all three damage sizes are reported in Table 5 and Table 6. Over the 200 samples acquired at the unseen positions, the mean deviation between the predicted and the ground-truth damage centre is 0.185 mm, with a standard deviation of 0.255 mm and a maximum of 2.071 mm. The mean absolute deviation of the damage diameter is 0.383 mm, with a standard deviation of 0.482 mm and a maximum of 1.698 mm. Compared with the results obtained on the 200 training-grid samples (mean centre deviation 0.133 mm, mean absolute diameter deviation 0.188 mm), a 10 mm offset from the training grid increases the mean centre deviation by 0.052 mm and the mean absolute diameter deviation by 0.195 mm, and both remain far below the smallest damage radius of 2.5 mm. The size estimation therefore degrades slightly more than the localization when the damage is moved away from the training grid, which is consistent with the fact that the diameter is inferred from the area of the reconstructed region and is thus more sensitive to a change in the spatial sampling. These results show that DARF-Net does not memorise discrete grid positions but learns the general correlation between the guided-wave response and the spatial position of the damage, and that it retains its imaging capability at positions that were not seen during training, within the range of offsets examined in this study (10 mm).

The measurements in this section were acquired with the mass-block surrogate; the extension to real impact damage is subject to the limitation discussed in Section 5.

#### 4.3.4. Model Comparison

To validate the comprehensive advantages of DARF-Net, four baselines are selected, covering two categories. The first category consists of three generic semantic segmentation models: Fast-SCNN [29], a lightweight real-time segmentation network with a multi-scale dual-branch structure; RepVGG [30], an efficient CNN based on structural re-parameterization; and DenseNet [31], a densely connected network. These three models represent lightweight real-time segmentation, structural re-parameterization and dense feature reuse, respectively. The second category is a domain-specific baseline, namely the Bayesian neural network proposed by Luo et al. [21] for ultrasonic Lamb-wave damage detection in CFRP composites, whose probabilistic modelling was reported to improve the robustness to noise and artifacts; in this work its Bayesian approximation is realized by Monte Carlo dropout, and the prediction is taken as the mean of ten stochastic forward passes. The domain-specific baseline is included because a comparison restricted to generic architectures does not fully position the proposed method within the ultrasonic imaging literature. All baselines use 6-channel images concatenated from the 0° and 90° data as input and output a 256 × 256 damage probability map, and the training strategy, data split and evaluation metrics are identical to those of DARF-Net, which ensures the fairness of the comparison. Besides the accuracy metrics, Table 7 reports the number of trainable parameters, the computational cost in FLOPs and the end-to-end inference time of every model. The parameter count indicates the model capacity, so that a gain can be attributed to the architectural design rather than to a larger network; the FLOPs quantify the computational cost of a single forward pass, computed as twice the number of multiply–accumulate operations for an input of 1 × 3 × 256 × 256 per view; and the latency is measured as input preparation plus network forward pass plus morphological post-processing on the target laptop with a batch size of one, after five warm-up runs and averaged over one hundred runs. Table 7 summarizes the quantitative evaluation results of each model on the test set, and Figure 14 compares their capabilities.

The three columns added to Table 7 separate the contribution of the architecture from that of the model capacity and of the inference cost. DARF-Net contains 5.50 M parameters, about five times fewer than DenseNet (27.56 M), which nevertheless reaches a lower F1 score (0.9138 versus 0.9849) and a lower recall (0.8448 versus 0.9800); Fast-SCNN and RepVGG, which are considerably smaller, also remain below DARF-Net in every metric. The domain-specific Bayesian baseline has 12.23 M parameters, i.e., more than twice those of DARF-Net, and reaches an F1 score of 0.9614, which is also below that of the proposed network. The improvement in detection performance is therefore not obtained by increasing the model capacity but by the architectural design, namely the dual-branch independent encoding of the two orthogonal views together with the artifact-resistant reconstruction path. The computational cost follows the same trend: the FLOPs increase from 5.40 G for Fast-SCNN and 9.47 G for RepVGG to 22.56 G for the Bayesian baseline, 25.03 G for DARF-Net and 46.11 G for DenseNet, so DARF-Net attains the highest accuracy at about half the computational cost of DenseNet. The measured end-to-end latency is 6.35 ms for Fast-SCNN, 6.99 ms for RepVGG, 18.48 ms for DenseNet, 34.58 ms for the Bayesian baseline and 14.79 ms for DARF-Net. The Bayesian baseline is the slowest of all models, because its inference rule requires ten Monte-Carlo samples for a single image, whereas DARF-Net completes the whole pipeline, including the morphological post-processing, in 14.79 ms. The two lightest generic models are faster than DARF-Net, but their recall of 0.5755 and 0.7033 means that between 28% and 41% of the true damages are missed, which is not acceptable for a damage-localization task. DARF-Net therefore offers the best trade-off among detection accuracy, computational cost and inference latency.

**Figure 15:**
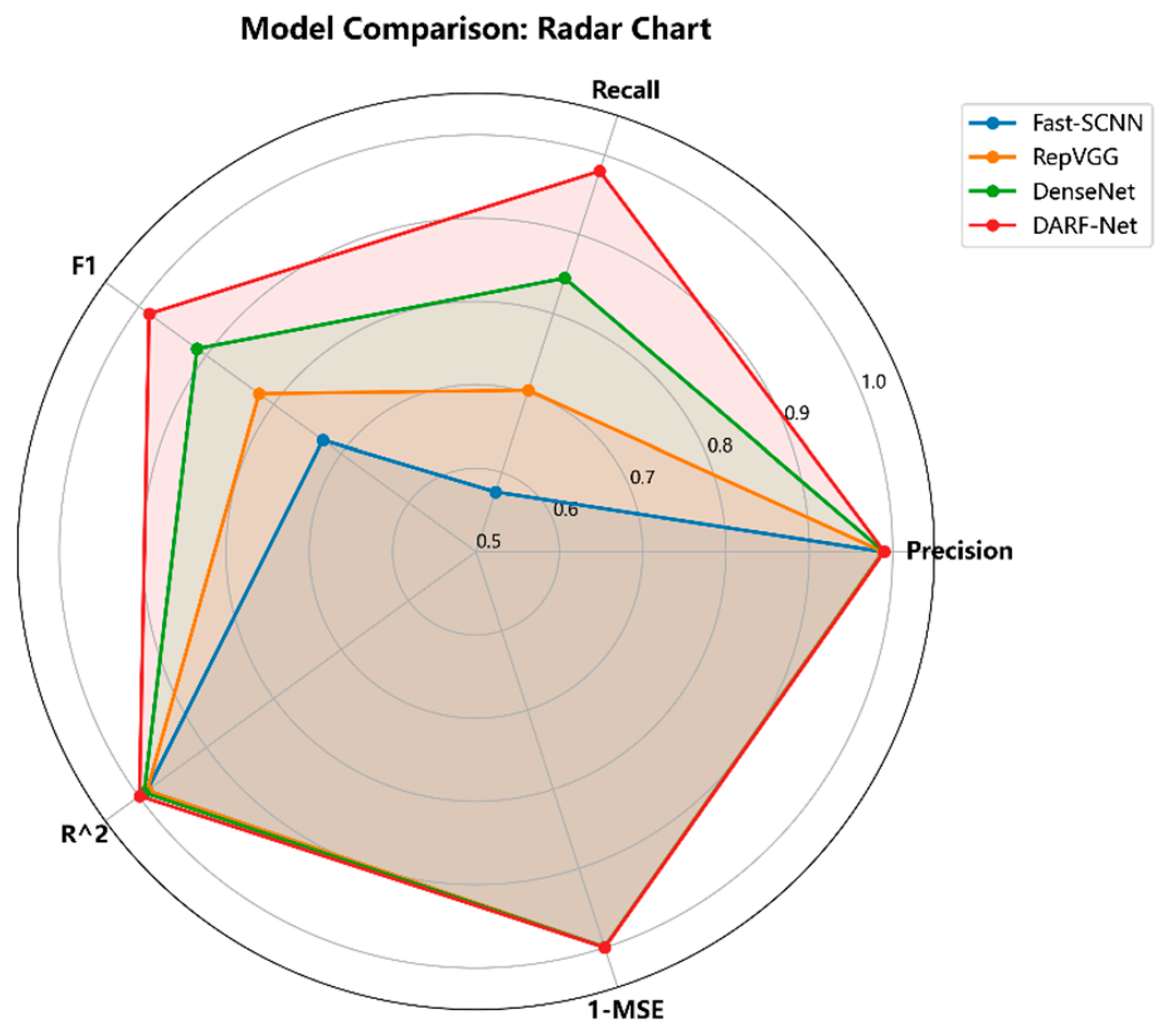
Radar chart for comparison of different models.

Table 7 and Figure 15 show the results. DARF-Net significantly outperforms the four comparison models in all metrics. Regarding pixel-level reconstruction accuracy, DARF-Net achieves an MSE of 0.000055. This is about 88.4% lower than the best baseline, DenseNet (0.000475). Its R^2^ reaches 0.9991. This indicates that the model’s ability to distinguish damage regions from the background far exceeds that of the comparison models.

Regarding region detection, the recall of the three generic models is significantly lower than that of DARF-Net: Fast-SCNN reaches only 0.5755, RepVGG 0.7033 and the best-performing generic model, DenseNet, 0.8448, so that many true damages are missed. The domain-specific Bayesian baseline reaches a recall of 0.9750 and an F1 score of 0.9614, which confirms that modelling the physical nature of guided-wave damage is more effective than transferring a generic segmentation architecture to this task; DARF-Net nevertheless remains higher on both metrics, so its advantage over the domain-specific baseline originates from the dual-branch independent encoding and the artifact-resistant reconstruction path rather than from the domain-specific design alone.

The above comparison experiments validate the core advantages of DARF-Net from an architectural perspective. First, dual-branch independent encoding fully exploits the complementary information from the 0° and 90° orthogonal views. It effectively addresses the insufficient information problem of single-direction scanning; the combination of CBAM attention and residual blocks enhances the network’s sensitivity to weak damage signals. It significantly improves recall. Third, morphological post-processing at the inference stage effectively suppresses false detections while maintaining high recall. It achieves synergistic optimization of precision and recall. Overall, DARF-Net demonstrates significant superiority over general semantic segmentation architectures in CFRP damage imaging tasks.

#### 4.3.5. Ablation Study

**Figure 16:**
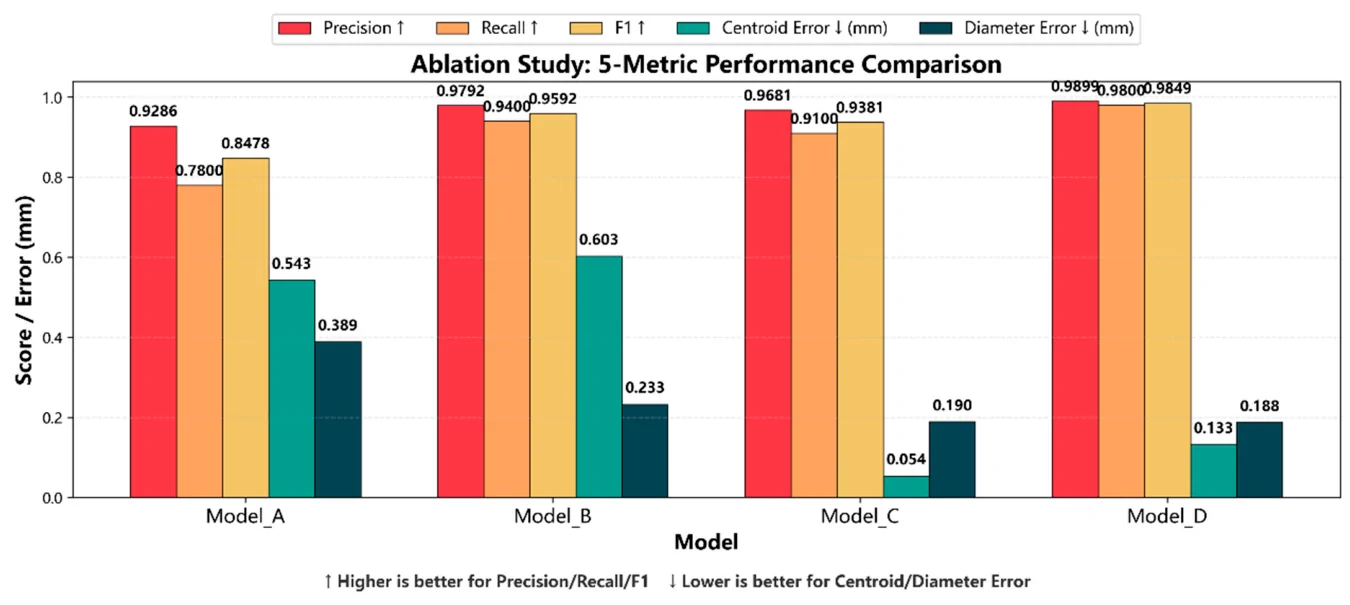
Bar chart for performance comparison of ablation models.

To further validate the effectiveness of the attention module and the residual block, four ablation models are designed. These are Model_A (no attention + no residual), Model_B (attention + no residual), Model_C (no attention + residual), and Model_D (attention + residual). All models adopt the same dual-branch encoder structure. They differ only in the presence or absence of the attention module and the residual block. In the testing stage all configurations are evaluated under one single protocol: the same training data and training budget, the same 200 test samples drawn with a fixed seed, the same binarisation threshold, the same connected-component matching criterion and the same morphological post-processing. The ablation covers three aspects. First, the encoding of the two views: the two encoders use independent parameters, and this choice is compared with a configuration in which they share their weights (a Siamese arrangement). Second, the fusion rule: element-wise addition is compared with channel concatenation and with attention-weighted fusion. Third, the morphological post-processing: the trained network is evaluated both with and without the post-processing, so that the contribution of the decision-level module is isolated. The sensitivity of the three geometric thresholds is reported in Table 8: the F1 score changes by less than 2% when the area threshold is swept from 40 to 160 pixels and by less than 1% when the aspect-ratio threshold is swept from 1.2 to 3.0, while only the solidity threshold is critical, with a 17% drop at S_min = 0.9 where genuine damages begin to be discarded. A single-direction input is not included as a performance baseline, because in this measurement configuration it is not a degraded variant of the method but a configuration in which the damage position is not observable: one line scan resolves the coordinate along the scanning line while the coordinate perpendicular to it is integrated over the aperture and therefore carries no positional information, so that the localization error along the unconstrained axis is undefined rather than merely large. Figure 16 uses a bar chart to intuitively compare the differences among the models. These differences are in three aspects: region detection, center positioning, and size estimation.

Figure 16 shows the results. Model_D achieves the highest precision, recall, and F1-score. Its recall is significantly better than that of the other models. It has the lowest missed detection rate. Model_C has the best center positioning accuracy. However, the mean center positioning deviation of Model_D is also extremely small. Furthermore, Model_C is inferior to Model_D in both region detection and size estimation. Model_D achieves the optimal comprehensive balance across three dimensions. These dimensions are damage detection, localization, and quantification. Therefore, this paper selects it as the final experimental model.

Table 9 assembles the ablations of the three components that carry the novelty of the method. Removing the morphological post-processing does not change the localization behaviour but has a clear effect on the decision: the F1 score decreases from 0.9849 to 0.9434 and the recall from 0.9800 to 0.9146, because the isolated artifact responses that the module removes would otherwise be counted as detections. Among the fusion rules, channel concatenation is the least accurate, with an F1 score of 0.8549 and a recall of 0.8100, whereas attention-weighted fusion reaches 0.9372; element-wise addition remains the best with an F1 score of 0.9849, and it is also the simplest rule, since it keeps the bottleneck at 512 channels, whereas channel concatenation doubles the bottleneck width to 1024 channels and attention-weighted fusion introduces an additional gating sub-network. Sharing the encoder weights is clearly detrimental: the F1 score drops to 0.6161 and the recall to 0.7100, because the two acquisition channels differ in their noise floor, gain drift and artifact orientation, so that a single set of filters cannot represent both. Taken together, the three components act on different aspects of the problem—artifact suppression at the decision level, view combination at the feature level, and channel-specific feature learning at the encoder level—and all three are required to reach the reported performance.

The variation in the F1 score is expressed relative to the value obtained at the default setting (A_min = 80 pixels, γ_max = 1.5, S_min = 0.8). The two shape-related thresholds can be varied over a range that is much wider than the values adopted here without changing the F1 score by more than 2%, whereas the solidity threshold is stable only up to 0.8: at S_min = 0.9 the recall falls from 0.355 to 0.275 because densely filled but slightly irregular damages begin to be discarded.

Table 9 reports the sensitivity of the three geometric thresholds. Each threshold is swept while the other two are held at the values adopted in this work (A_min = 80 pixels, γ_max = 1.5 and S_min = 0.8), and the vertical axis gives the change relative to this default setting. The F1 score changes by less than 2% when the area threshold is swept from 40 to 160 pixels and by less than 1% when the aspect-ratio threshold is swept from 1.2 to 3.0, which shows that these two thresholds only have to separate isolated noise clusters and elongated artifacts from compact damages, a separation that they achieve over a wide interval. The solidity threshold is the only one to which the result is sensitive: the F1 score remains within ±0.6% for S_min between 0.4 and 0.8 and falls by about 17% at S_min = 0.9, where the recall drops sharply because densely filled but slightly irregular damages begin to be discarded.

## 5. Conclusions and Future Work

To meet the demand for non-contact damage imaging of CFRP, this paper proposes an air-coupled ultrasonic guided wave imaging method. The method is based on a dual-branch convolutional neural network. It constructs an end-to-end reconstruction framework with dual inputs of 0° and 90°. The dual-branch encoder effectively extracts complementary features from two views. Residual blocks in the decoder improve reconstruction details. Ablation studies and model comparison experiments validate the effectiveness of the design. They do so at the module level and architecture level, respectively. Overall, DARF-Net combines dual-branch cross-localization and an attention mechanism. This synergy effectively solves the missed detection problem in single-view imaging. This problem is caused by anisotropy. It provides a solution for rapid air-coupled ultrasonic imaging of CFRP damage. This solution balances both accuracy and efficiency.

Although the proposed method achieves relatively promising results, the experiments have limitations. The experiments only cover single-damage scenarios of three fixed sizes. The damage is represented by mass blocks coupled to the plate rather than by a real low-velocity impact. Although this surrogate prescribes the damage diameter exactly and allows the same specimen to be reused at all grid positions, it does not reproduce the through-thickness morphology of a delamination nor the multiple scattering inside the delaminated interface, so the reported localization and sizing accuracies are valid for surrogate damage of the three calibrated sizes. Validation on real low-velocity impact damage, with ultrasonic C-scan as the independent reference measurement, is therefore the first direction of future work.

Future work will focus on several aspects. First, it will expand the diversity of damage types and sizes. Second, it will conduct experimental validation on different materials and structures. Third, it will explore lightweight network structures and transfer learning strategies. These efforts aim to advance the method toward practical engineering applications.

## Figures and Tables

**Table 1:** Parameters of the CFRP plate.

E_1_/GPa	E_2_/GPa	E_3_/GPa	G_12_/GPa	G_13_/GPa	G_23_/GPa	ν_12_	ν_13_	ν_23_	ρ/(kg/m^3^)
120	8	8	3.24	3.24	2.433	0.28	0.28	0.366	1550

**Table 2:** Evaluation metrics of training performance.

Epoch	MSE	RMSE	MAE	R^2^
60	0.000055	0.007407	0.001795	0.999101

**Table 3:** Statistics of Center Positioning Deviation.

Number of Samples	Mean Deviation (mm)	Standard Deviation (mm)	Maximum Deviation (mm)
200	0.133	0.247	1.194

**Table 4:** Statistics of Damage Diameter Deviation.

Number of Samples	Mean Absolute Deviation (mm)	Standard Deviation (mm)	Maximum Deviation (mm)
200	0.188	0.501	1.875

**Table 5:** Statistics of centre positioning deviation at the 16 non-training positions.

Number of Samples	Mean Deviation (mm)	Standard Deviation (mm)	Maximum Deviation (mm)
200	0.185	0.255	2.071

**Table 6:** Statistics of damage diameter deviation at the 16 non-training positions.

Number of Samples	Mean Absolute Deviation (mm)	Standard Deviation (mm)	Maximum Deviation (mm)
200	0.383	0.482	1.698

**Table 7:** Comparative experimental results of different models.

Model	Params (M)	FLOPs (G)	Latency (ms)	MSE	R^2^	Precision	Recall	F1
Fast-SCNN	**0.45**	**5.40**	**6.35**	0.000750	0.9877	0.9882	0.5755	0.7274
RepVGG	2.55	9.47	6.99	0.000736	0.9879	0.9884	0.7033	0.8218
DenseNet	27.56	46.11	18.48	0.000475	0.9922	0.9893	0.8448	0.9138
BNN	12.23	22.56	34.58	0.000142	0.9983	0.9299	0.9750	0.9614
DARF-Net	5.50	25.03	14.79	**0.000055**	**0.9991**	**0.9899**	**0.9800**	**0.9849**

**Table 8:** Sensitivity of the three geometric thresholds.

Threshold	Range Swept	Variation in F1	Observation
A_min (pixels)	40–160	−2.0% to 0.0%	insensitive over the whole range
γ_max	1.2–3.0	−1.0% to 0.0%	insensitive over the whole range
S_min	0.4–0.9	−16.8% to +0.6%	stable for 0.4–0.8; −17% at 0.9

**Table 9:** Ablation study of the proposed network. (Note: the first two rows are obtained by evaluating the trained network with and without the morphological post-processing; the remaining rows correspond to variants that were retrained from scratch under the same protocol.)

Configuration	Precision	Recall	F1
DARF-Net (proposed)	0.9899	0.9800	0.9849
w/o morphological post-processing	0.9740	0.9146	0.9434
Fusion: channel concatenation	0.9050	0.8100	0.8549
Fusion: attention-weighted	0.9835	0.8950	0.9372
Encoder: shared weights (Siamese)	0.5441	0.7100	0.6161

## Data Availability

The data presented in this study are available on request from the corresponding author. The raw guided-wave signals and the corresponding label images were acquired with a custom air-coupled ultrasonic scanning system and are not publicly archived, because their release would compromise the proprietary configuration of the measurement system and the effort invested in the experimental campaign. Processed data supporting the reported results can be provided to qualified researchers for non-commercial academic use.

## Abbreviations

The following abbreviations are used in this manuscript:

CFRP	Carbon fiber reinforced polymer
NDT	Nondestructive testing
DARF-Net	Dual-branch Artifact-Resistant Fast Imaging Network
SNR	Signal-to-noise ratio
CBAM	Convolutional Block Attention Module
MSE	Mean squared error
RMSE	Root mean square error
MAE	Mean absolute error
